# The Dominant-Subthalamic Nucleus Phenomenon in Bilateral Deep Brain Stimulation for Parkinson’s Disease: Evidence from a Gait Analysis Study

**DOI:** 10.3389/fneur.2017.00575

**Published:** 2017-10-30

**Authors:** Mario Giorgio Rizzone, Maurizio Ferrarin, Michele Maria Lanotte, Leonardo Lopiano, Ilaria Carpinella

**Affiliations:** ^1^Department of Neuroscience Rita Levi Montalcini, University of Turin, Turin, Italy; ^2^Biomedical Technology Department, IRCCS Don Carlo Gnocchi Foundation, Milan, Italy

**Keywords:** Parkinson’s disease, deep brain stimulation, dominant subthalamic nucleus, gait, instrumented movement analysis, kinematics, kinetics, electromyography

## Abstract

**Background:**

It has been suggested that parkinsonian [Parkinson’s disease (PD)] patients might have a “dominant” (DOM) subthalamic nucleus (STN), whose unilateral electrical stimulation [deep brain stimulation (DBS)] could lead to an improvement in PD symptoms similar to bilateral STN-DBS.

**Objectives:**

Since disability in PD patients is often related to gait problems, in this study, we wanted to investigate in a group of patients bilaterally implanted for STN-DBS: (1) if it was possible to identify a subgroup of subjects with a dominant STN; (2) in the case, if the unilateral stimulation of the dominant STN was capable to improve gait abnormalities, as assessed by instrumented multifactorial gait analysis, similarly to what observed with bilateral stimulation.

**Methods:**

We studied 10 PD patients with bilateral STN-DBS. A clinical evaluation and a kinematic, kinetic, and electromyographic (EMG) analysis of overground walking were performed—off medication—in four conditions: without stimulation, with bilateral stimulation, with unilateral right or left STN-DBS. Through a hierarchical agglomerative cluster analysis based on motor Unified Parkinson’s Disease Rating Scale scores, it was possible to separate patients into two groups, based on the presence (six patients, DOM group) or absence (four patients, NDOM group) of a dominant STN.

**Results:**

In the DOM group, both bilateral and unilateral stimulation of the dominant STN significantly increased gait speed, stride length, range of motion of lower limb joints, and peaks of moment and power at the ankle joint; moreover, the EMG activation pattern of distal leg muscles was improved. The unilateral stimulation of the non-dominant STN did not produce any significant effect. In the NDOM group, only bilateral stimulation determined a significant improvement of gait parameters.

**Conclusion:**

In the DOM group, the effect of unilateral stimulation of the dominant STN determined an improvement of gait parameters similar to bilateral stimulation. The pre-surgical identification of these patients, if possible, could allow to reduce the surgical risks and side effects of DBS adopting a unilateral approach.

## Introduction

Subthalamic nucleus (STN) deep brain stimulation (DBS) is currently a widely performed procedure for the treatment of advanced Parkinson’s disease (PD). Bilateral STN-DBS has been demonstrated as an effective treatment for advanced PD patients both in the short and in the long term, leading to a good control of all the PD cardinal symptoms (rigidity, bradykinesia, and tremor) and of the drug-induced motor complications (motor fluctuations and dyskinesias) (1–7). Moreover, studies performed with instrumented movement analysis showed the significant improvements provided by bilateral STN-DBS on anticipatory postural adjustments before gait initiation (8), kinematics and kinetics of lower limb joints during steady-state walking (9), and upper limb locomotor synergies (10), that are commonly affected in the advanced stage of PD.

Even though bilateral STN-DBS is considered a relatively safe procedure, in the last years there has been a growing interest in unilateral and staged STN-DBS (i.e., implant of the two electrodes during two distinct surgical sittings separated over time), which involves lower surgical risk and less post-operative complications, such as cognitive dysfunctions (11–14). Alberts et al. (13), for example, showed a decline in cognitive and motor functions when patients were examined under dual-task conditions only with bilateral but not with unilateral STN-DBS. In addition, several studies demonstrated that also unilateral STN-DBS is effective in the control of PD symptoms, with an improvement in Unified Parkinson’s Disease Rating Scale (UPDRS) (15) total scores ranging from 20 to 40% (16–20). The improvement of PD motor symptoms obtained by unilateral STN-DBS is mainly observed in the body side contralateral to the stimulated STN; nevertheless, several papers also demonstrated a relevant ipsilateral effect of unilateral STN-DBS (21–23). Moreover, the possible dominance of right-side motor networks for gait control has been hypothesized in a recent paper by Lizarraga et al. (24), which addressed the effects of bilateral, right- or left-side DBS on gait.

Castrioto et al. (25) demonstrated that, in a relevant percentage of PD patients treated by bilateral STN-DBS, it was possible to identify a so called “dominant STN,” whose unilateral stimulation was capable to determine a clinical improvement similar to bilateral stimulation. The authors showed the presence of a dominant STN in 50% of patients; in about 73% of the cases the dominant STN was contralateral to the most affected side of the body, while in the others it was ipsilateral. In the dominant-STN group, the percentage of improvement of motor UPDRS was 41.7% with bilateral STN-DBS and 35.4% with unilateral dominant STN-DBS, while in the non-dominant STN group a significant improvement in the motor UPDRS score was found only with bilateral stimulation. This finding has increased the interest of unilateral STN-DBS as a possible alternative approach to bilateral DBS in some PD patients.

In the perspective to identify PD patients with a dominant STN before surgery, the authors suggested the need of further studies (25).

Gait disorders play an outstanding role in PD, becoming with the progression of disease one of the major source of disability (26–28). Unfortunately, the ability to detect gait changes through the UPDRS gait item is limited.

On the basis of these considerations, the aim of the present work was to investigate in a group of PD patients bilaterally implanted for STN-DBS: (1) if it was possible to identify a subgroup of patients with a dominant STN with the method indicated by Castrioto et al.; (2) in the case, if the unilateral stimulation of the dominant STN was capable to improve gait anomalies similarly to what observed with bilateral stimulation, using a kinematic, kinetic, and electromyographic (EMG) analysis of overground walking.

## Materials and Methods

### Subjects

Ten patients with idiopathic PD, bilaterally implanted in the STN as described in Lopiano et al. (29), and 10 age-matched healthy controls (5 males, 5 females; mean ± SD age 61.4 ± 5.0; age range 55–69 years) voluntarily took part in the study. The main characteristics of PD patients were the following: five males, five females; age (mean ± SD): 60.2 ± 4.8 years (range 52–68 years); PD duration: 16.9 ± 5.5 years; time postsurgery: 10.4 ± 7.0 months; and Hoehn and Yahr stage in medication off: 3.7 ± 0.7. The inclusion criteria for surgery had been a diagnosis of idiopathic PD with severe motor fluctuation or dyskinesias, a good response to levodopa challenge (UPDRS motor score improvement ≥30%), the absence of relevant cognitive or psychiatric disturbances and the absence of significant abnormalities at the brain imaging (MRI). The mean levodopa dosage at the time of the study was 112.5 ± 186.1 mg/day (range 0–500 mg/day), and the mean levodopa equivalent dosage was 274.5 ± 303.5 mg/day (range 0–950 mg/day); three patients were without antiparkinsonian drugs.

All patients were evaluated preoperatively by the UPDRS III (motor section) in the off condition (overnight withdrawal of all antiparkinsonian drugs) and in the on condition (about 60 min after the administration of a levodopa dose 25% higher of the first morning dose). UPDRS IV (complications of therapy) was also administered.

After surgery, the position of the active contacts of the electrodes was calculated as described in a previous paper (30).

All subjects gave their written informed consent to the experimental protocol, which conformed to the Declaration of Helsinki and was approved by the Ethics Committee of CTO Hospital (Turin).

### Experimental Protocol

After a 12-h washout of all antiparkinsonian drugs, patients performed four sets of walking trials in the following conditions: (1) bilateral STN stimulation (BIL); (2) right STN stimulation (UNI_R); (3) left STN stimulation (UNI_L); (4) stimulation off (OFF). BIL was the chronic condition of the patients as they arrived to the lab and it was always tested first to minimize the duration of the whole session by avoiding initial and posttrial washout phases. After the assessment under BIL stimulation, conditions UNI_R, UNI_L and OFF were randomized among patients. Patients were always unaware of the current condition. After each change of stimulation parameters there was a resting time of at least 60 min, which was considered enough for DBS washout (31).

For each condition, subjects performed eight walking trials at their preferred speed along a 10 m path. Subjects with PD were evaluated by the UPDRS motor section (part III) before each gait session; the sub-scores for tremor (items 20, 21), rigidity (item 22), akinesia (items 23, 24, 25, 26, and 31), gait (item 29), postural stability (item 30), and the separate scores for right and left sides of the body were also calculated.

The duration of the entire experimental procedure, including subjects’ preparation, instrumental gait sessions, clinical assessments, and resting periods, was about 5 h and a half.

Non-disabled controls underwent only one gait analysis session consisting of eight walking trials at self-selected speed.

### Setup

Kinematics of body segments were measured during walking, using an optoelectronic system (ELITE, BTS, Milan, Italy—four cameras, sampling frequency 50 Hz), which computed the 3D coordinates of spherical markers (10 mm diameter) fixed on the following bony landmarks: sacrum, posterior superior iliac spines, lateral femoral condyles, lateral malleoli, and fifth metatarsal heads of both sides of the body. Ground reaction forces were acquired at a sampling frequency of 50 Hz with a dynamometric platform (Kistler, GmbH, Winterthur, Switzerland). Surface EMG signals were recorded using a telemetric 8-channel system (TELEMG, BTS, Milan, Italy) from tibialis anterior (TA), gastrocnemius medialis (GAM), rectus femoris (RF), and semimembranosus (SM) muscles of both legs. Myoelectric signals were collected by preamplified Ag/AgCl electrodes (diameter: 25 mm, bipolar configuration, interelectrode distance: 20 mm), band-pass filtered (between 10 and 200 Hz), and acquired at a sampling frequency of 500 Hz.

### Data Processing

Markers’ coordinates and ground reactions were processed to produce hip, knee and ankle joint angles, moments, and power in the sagittal plane. The time course of all kinematic and kinetic variables were time normalized as a percentage of the stride duration (between two consecutive heel strike of the same foot), and for each cycle the following variables were computed: spatiotemporal gait parameters (walking velocity, cadence, stride length, and stance phase duration), range of motion (ROM) and moment and power peak of hip, knee, and ankle joints. EMG signals were high-pass filtered at 50 Hz (fourth order zero-lag Butterworth filter) to remove movement artifacts and then they were rectified and low-pass filtered at 7.5 Hz to obtain linear envelopes (32). All EMG profiles were normalized in time as a percentage of the stride duration. Finally, to quantify the level of motor output, the root mean square (RMS) of each envelope EMG signal was computed over functionally relevant stride periods, in particular: first double stance for TA and RF, push-off phase for GAM, early swing for TA and late swing for SM. No amplitude normalization was applied since RMS values were compared within subjects among the four STN stimulation conditions, without removal and replacement of the electrodes.

### Statistical Analysis

A hierarchical agglomerative cluster analysis was performed, following the method used by Castrioto et al. (25), to determine whether the motor UPDRS score from different stimulation conditions supported the existence of a group of PD subjects with a dominant STN. In particular, based on the effects of unilateral stimulation on clinical motor scores, it was possible to identify for each subject the most effective (UNI_best_) and the least effective (UNI_worst_) side of stimulation. Subsequently, six ratios between scores related to different stimulation conditions were computed: UNI_best_/BIL, UNI_worst_/BIL, OFF/BIL, UNI_worst_/UNI_best_, UNI_best_/OFF, UNI_worst_/OFF. The cluster analysis was performed on these six variables using the correlation distance measure and the McQuitty (Weighted Pair Group Method with Arithmetic Mean) agglomeration method. On the basis of the results of this analysis, PD subjects were subsequently pooled into two groups, according to the presence (DOM group) or not (NDOM group) of a dominant STN.

Considering the small sample size, data were analyzed using non-parametric tests. Differences among stimulation conditions were analyzed using Friedman test and Tukey–Kramer *post hoc* test. Differences in UPDRS III scores and gait analysis variables between stimulation sides and body sides were evaluated by Wilcoxon matched pairs test (Wt). The same test was used for the comparison of stimulation parameters and position of the electrodes between right and left side. Comparisons between DOM and NDOM groups and between each PD group and controls were performed using Mann–Whitney *U* test (MWt). Level of significance was set to 0.05.

## Results

### Cluster Analysis

**Figure 1:**
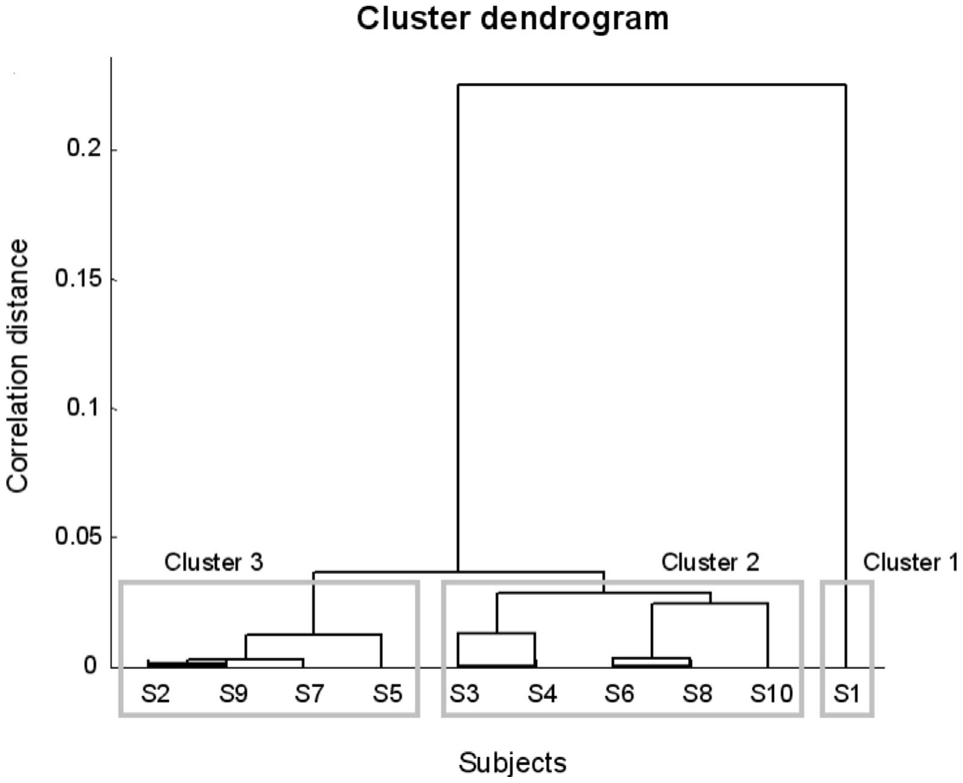
Dendrogram representing the subgroups of Parkinson’s disease subjects derived from the cluster analysis.

Three clusters were identified (Figure 1). The first cluster included one subject (S1). In this patient, right STN stimulation alone achieved an improvement of UPDRS III score equal to that obtained with bilateral STN stimulation and 50% greater than that obtained with left stimulation.

The second cluster included five subjects (S3, S4, S6, S8, and S10) where the unilateral stimulation of one side (right side for S3, S4, and S8; left side for S6 and S10) induced a mean motor improvement 11% higher than that obtained stimulating the other side and 16% smaller than that achieved by bilateral stimulation.

The third cluster included four patients (S2, S5, S7, and S9) where unilateral right and left stimulation produced similar mean improvements (mean difference 2%), 30% smaller than those generated by bilateral stimulation.

In summary, 6 of the 10 tested patients (DOM group, 60%) showed the presence of a dominant STN, whose stimulation produced motor improvements equal or slightly inferior to bilateral stimulation. Four of these subjects demonstrated right dominance, whereas two patients showed left dominance. In one subject (S10), the dominant STN was ipsilateral to the most affected side. Conversely, 4 of the 10 tested subjects (NDOM group, 40%) did not show the presence of a dominant STN and obtained greater improvements upon bilateral stimulation.

### Dominant-STN Group versus Non-Dominant STN Group

#### Presurgical Evaluations

As reported in Table 1, the dominant-STN (DOM) and the non-dominant STN (NDOM) groups showed similar demographic characteristics, even though there was a trend toward a longer disease duration for the DOM group (*p*_MWt_ = 0.085). The disease severity was similar in the two groups, except for a significantly lower postural stability (*p*_MWt_ = 0.040) and poorer gait function (*p*_MWt_ = 0.040) of the DOM group in the medication-off condition, as indicated by the specific UPDRS items. Moreover, in medication-on condition, the DOM group demonstrated more therapy-related complications than NDOM group (*p*_MWt_ = 0.014).

**Table 1:** Demographic clinical characteristics (median and range) for Parkinson’s disease (PD) subjects with a dominant subthalamic nucleus (STN) (DOM) and PD subjects without a dominant STN (NDOM).

	DOM (*n* = 6)	NDOM (*n* = 4)	*p* (MWt)
Age (years)	59.0 (52.0–68.0)	61.0 (57.0–66.0)	0.593
Gender (no. male/no. female)	2/4	3/1	
Disease duration (years)	21.0 (13.0–22.0)	13.5 (8.0–17.0)	0.085
Time from surgery (months)	11.0 (8.0–28.0)	8.0 (4.0–9.0)	0.234
H&Y stage (medication and stimulation off)	4.0 (2.5–4.5)	3.5 (3.0–4.0)	0.581
**Presurgery (medication off)**			
UPDRS III Motor Examination	62.0 (46.0–95.0)	57.8 (44.5–73.0)	0.522
Tremor (items 20, 21)	6.5 (2.0–22.5)	9.8 (4.0–17.0)	0.394
Rigidity (item 22)	12.8 (10.5–19.5)	12.8 (8.0–17.5)	0.522
Akinesia (items 23, 24, 25, 26, 31)	26.8 (21.0–32.0)	23.8 (21.0–26.0)	0.278
Postural stability (item 30)	2.5 (2.0–4.0)	2.0 (1.0–2.0)	**0.040**
Gait (item 29)	2.5 (2.0–4.0)	2.0 (1.0–2.0)	**0.040**
Asymmetry of symptoms^a^	3.8 (1.1–11.9)	5.7 (2.7–9.3)	0.286
**Presurgery (medication on)**			
UPDRS III Motor Examination	21.0 (10.0–36.5)	21.3 (5.5–22.0)	0.522
Tremor (items 20, 21)	0.0 (0.0–1.0)	0.5 (0.0–2.0)	0.456
Rigidity (item 22)	5.0 (2.0–8.5)	5.3 (0.0–7.5)	0.831
Akinesia (items 23, 24, 25, 26, 31)	8.5 (1.0–17.0)	8.0 (2.0–9.0)	0.594
Postural stability (item 30)	1.8 (1.0–3.0)	1.5 (1.0–2.0)	0.594
Gait (item 29)	0.8 (0.0–3.0)	0.5 (0.5–0.5)	0.394
Asymmetry of symptoms^a^	5.8 (1.4–36.4)	4.5 (0.0–19.5)	0.640
**Presurgery (complications of therapy)**			
UPDRS IV (items 32, 33, 39)	6.3 (4.0–7.0)	3.0 (3.0–4.0)	**0.014**

*^a^Asymmetry of symptoms: % [UPDRS (worst side) − UPDRS (best side)]/total UPDRS III*.

*UPDRS, Unified Parkinson’s Disease Rating Scale; H&Y, Hoehn and Yahr stage; MWt, Mann–Whitney U test*.

*Significant differences between DOM and NDOM group (*p* < 0.05) are reported in bold*.

#### Effects of STN Stimulation on Clinical Motor Symptoms

Results related to motor UPDRS III are reported in Table 2 (A).

**Table 2:** Median (range) values of postsurgery clinical characteristics (A) and quantitative gait parameters (B–E) for Parkinson’s disease (PD) patients with dominant subthalamic nucleus (STN) (DOM) and PD patients without dominant STN (NDOM) in the different conditions.

	Control (*n* = 10)	DOM (*n* = 6)				NDOM (*n* = 4)			
		OFF^(a)^	UNI_ND^(b)^	UNI_D^(c)^	BIL^(d)^	OFF^(a)^	UNI_R^(b)^	UNI_L^(c)^	BIL^(d)^
**A. Clinical variables**									
UPDRS III total		60.8^(c,d)^ (42.0–80.5)	39.5^(d)^ (21.0–51.5)	26.8^(a)^ (17.5–39.0)	16.3^(a,b)^ (7.5–29.0)	62.5^(d)^ (60.5–72.0)	47.0 (36.0–54.0)	47.5 (34.5–53.0)	28.3^(a)^ (10.5–36.5)
Tremor (items 20, 21)		4.5^(c,d)^ (1.5–13.0)	3.8 (0.0–9.5)	0.8^(a)^ (0.0–7.5)	0.3^(a)^ (0.0–3.5)	10.5^(d)^ (1.0–19.0)	5.8 (0.5–11.0)	6.8 (0.0–10.0)	2.0^(a)^ (0.0–5.5)
Rigidity (item 22)		15.5^(c,d)^ (11.0–19.0)	10.5 (6.5–15.0)	8.0^(a)^ (5.0–10.5)	5.0^(a)^ (2.0–7.5)	13.0^(d)^ (11.5–18.0)	10.3 (8.0–11.0)	11.5 (6.5–13.5)	7.8^(a)^ (2.0–9.5)
Akinesia (items 23, 24, 25, 26, 31)		25.5^(c,d)^ (15.0–31.0)	17.5 (3.5–21.0)	9.0^(a)^ (5.5–15.5)	4.0^(a)^ (2.0–12.0)	28.3^(d)^ (25.5–31.5)	21.5 (21.0–22.0)	20.0 (18.0–24.0)	11.3^(a)^ (6.5–14.0)
Postural stability (item 30)		2.0^(d)^ (1.5–3.0)	2.0 (1.0–2.0)	1.8 (1.0–2.0)	1.5^(a)^ (1.0–2.0)	1.8^(d)^ (1.0–3.0)	1.5 (0.5–2.0)	1.5 (0.5–2.0)	1.0^(a)^ (0.5–2.0)
Gait (item 29)		2.5^(d)^ (1.5–3.5)	1.5 (0.0–2.5)	1.3 (0.0–2.5)	0.5^(a)^ (0.0–1.0)	2.0^(d)^ (1.0–3.5)	1.5 (1.0–3.0)	1.3 (1.0–3.0)	0.8^(a)^ (0.0–1.5)

**B. Spatiotemporal gait variables**									
Velocity (% body height/s)	65.0 (45.8–86.3)	*30.3^(c,d)^ (11.3–41.5)	40.3 (28.1–65.1)	53.4^(a)^ (10.1–31.5)	54.2^(a)^ (39.1–67.8)	*35.7^(d)^ (21.6–57.9)	38.2 (26.8–67.7)	44.0 (27.4–66.9)	49.5^(a)^ (29.9–67.8)
Stride length (% body height)	74.1 (57.8–88.5)	*33.4^(c,d)^ (11.0–45.4)	40.6^(d)^ (27.1–64.7)	56.5^(a)^ (32.5–68.5)	62.2^(a,b)^ (46.5–73.5)	*48.0^(d)^ (33.0–57.9)	52.9 (42.3–66.6)	54.3 (43.4–65.2)	57.7^(a)^ (46.0–69.8)
Cadence (stride/min)	54.9 (47.5–60.5)	55.5 (38.5–61.8)	60.4 (48.0–77.6)	53.8 (40.4–64.5)	55.4 (43.4–61.1)	44.0 (38.9–60.09)	43.5 (36.7–61.0)	48.2 (37.8–61.6)	49.7 (39.0–60.7)
Stance time (% stride)	59.4 (55.5–62.3)	*64.9 (59.2–71.5)	62.7 (57.0–64.2)	59.5 (58.3–67.0)	60.2 (58.3–63.4)	*64.8 (59.4–68.9)	63.3 (58.6–68.3)	62.2 (59.2–68.5)	61.9 (58.8–70.3)

**C. Kinematic gait variables**									
Hip range of motion (ROM) (°)	50.8 (40.5–65.4)	*26.4^(c,d)^ (18.9–35.6)	31.0^(d)^ (20.1–43.7)	40.2^(a)^ (23.6–47.5)	44.8^(a,b)^ (32.9–52.1)	*33.7 (23.5–41.5)	39.9 (30.3–49.3)	37.2 (32.2–43.6)	42.9 (29.1–47.3)
Knee ROM (°)	59.2 (56.4–67.6)	*36.7^(c,d)^ (23.6–45.5)	44.8 (28.4–51.2)	48.9^(a)^ (26.9–61.4)	51.6^(a)^ (43.1–60.9)	*41.5 (29.8–51.2)	45.2 (33.7–61.8)	45.3 (37.7–52.1)	45.9 (38.3–54.9)
Ankle ROM (°)	26.1 (20.3–34.5)	*13.4^(c,d)^ (8.7–18.6)	16.1^(d)^ (14.4–20.0)	20.7^(a)^ (15.7–22.1)	22.5^(a,b)^ (19.8–23.7)	*16.7 (13.6–18.7)	18.4 (17.2–21.8)	18.1 (16.6–21.0)	17.9 (15.2–18.1)

**D. Kinetic gait variables**									
Hip moment peak (Nm/kg)	1.07 (0.63–1.60)	0.93 (0.59–1.26)	0.90 (0.53–1.41)	1.09 (0.50–1.38)	1.21 (0.63–1.37)	1.23 (0.61–1.52)	1.22 (1.07–1.48)	1.22 (0.62–1.42)	1.09 (0.59–1.45)
Knee moment peak (Nm/kg)	0.40 (0.16–0.85)	0.33^(d)^ (0.16–0.44)	0.41 (0.24–0.51)	0.45 (0.37–0.70)	0.59^(a)^ (0.18–0.81)	0.40 (0.12–0.70)	0.31 (0.23–0.80)	0.32 (0.24–0.85)	0.33 (0.30–0.46)
Ankle moment peak (Nm/kg)	1.52 (1.31–1.65)	*0.99^(c,d)^ (0.97–1.29)	1.16 (0.83–1.31)	1.25^(a)^ (1.07–1.31)	1.27^(a)^ (0.99–1.39)	1.34 (1.03–1.45)	1.22 (0.96–1.54)	1.20 (0.91–1.52)	1.29 (0.91–1.59)
Hip power peak (W/kg)	2.06 (0.93–2.67)	*0.78 (0.37–1.66)	1.15 (0.42–1.89)	1.84 (0.50–2.54)	1.92 (0.52–2.30)	1.32 (0.71–1.87)	1.55 (1.28–3.00)	1.51 (0.94–2.65)	1.21 (1.03–2.79)
Knee power peak (W/kg)	0.30 (0.13–0.93)	0.24 (0.07–0.45)	0.20 (0.11–0.49)	0.29 (0.09–0.75)	0.32 (0.23–0.90)	0.08 (0.07–0.53)	0.20 (0.06–0.98)	0.18 (0.06–0.84)	0.28 (0.06–0.34)
Ankle power peak (W/kg)	3.06 (1.63–4.59)	*0.86^(c,d)^ (0.47–1.60)	1.29 (0.80–2.98)	1.89^(a)^ (0.84–2.86)	2.03^(a)^ (1.25–3.05)	1.27 (0.69–4.81)	1.72 (0.57–3.02)	1.68 (0.60–4.64)	2.03 (0.70–2.48)

**E. Electromyographic root mean square**									
Semimembranosus (late swing) (μV)	23.7 (8.3–64.6)	19.1 (10.4–47.8)	23.2 (14.3–88.3)	24.6 (11.9–72.4)	26.4 (9.6–67.1)	22.0 (10.0–51.5)	21.2 (9.2–82.9)	24.6 (15.6–68.6)	31.2 (15.2–78.8)
Rectus femoris (first double stance) (μV)	25.1 (13.5–64.2)	*19.2 (10.4–30.6)	19.7 (11.9–66.2)	30.4 (9.2–59.4)	31.6 (11.8–60.3)	*14.3 (6.4–47.6)	15.2 (10.2–47.7)	25.1 (13.4–62.3)	28.4 (11.6–80.3)
Gastrocnemius medialis (push–off) (μV)	124.3 (36.0–232.0)	*33.5^(c,d)^ (6.2–88.0)	42.5^(d)^ (14.5–106.9)	47.4^(a)^ (12.0–148.4)	59.5^(a,b)^ (17.5–186.1)	*35.0^(b,d)^ (21.9–88.8)	47.7^(a)^ (30.1–97.1)	39.8 (17.5–93.8)	47.3^(a)^ (31.1–105.5)
Tibialis anterior (TA) (first double stance) (μV)	113.6 (51.0–148.8)	*23.6^(c,d)^ (10.6–69.6)	31.5^(d)^ (13.9–71.5)	51.1^(a)^ (18.9–136.4)	57.7^(a,b)^ (26.8–159.1)	*22.9^(d)^ (7.3–47.1)	25.0 (6.3–65.2)	17.0 (6.6–61.5)	37.7^(a)^ (7.5–67.6)
TA (early swing) (μV)	79.8 (36.5–165.1)	*52.9 (30.0–81.4)	64.8 (12.2–84.8)	55.0 (11.2–149.7)	55.7 (13.3–95.6)	*44.5 (18.8–138.7)	58.0 (17.4–140.7)	41.6 (21.2–11.5)	48.6 (21.1–126.9)

*Control values are also reported*.

*OFF, basal condition (medication off-bilateral stimulation off); UNI_D, unilateral stimulation of dominant STN; UNI_ND, unilateral stimulation of non-dominant STN; UNI_R, unilateral stimulation of right STN; UNI_L, unilateral stimulation of left STN; BIL, bilateral STN stimulation; UPDRS, Unified Parkinson’s Disease Rating Scale*.

*The superscript letters (a)–(d) indicate a significant difference between two specific conditions (*p* < 0.05, Friedman test and Tukey–Kramer *post hoc* test)*.

Symbol “*” indicates statistically significant differences between PD subjects in OFF condition and controls (*p* < 0.05, Mann–Whitney *U* test).

DOM and NDOM groups showed comparable UPDRS III scores in basal (OFF) condition (*p*_MWt_ > 0.324) and under bilateral STN stimulation (*p*_MWt_ > 0.114). In the DOM group, both bilateral STN stimulation (BIL) and unilateral stimulation of the dominant side (UNI_D) induced statistically significant improvements of UPDRS III total score and sub-scores related to tremor, rigidity, and akinesia, whereas unilateral stimulation of the non-dominant STN (UNI_ND) did not produce significant ameliorations with respect to basal condition (OFF). Postural stability and gait sub-scores, instead, appeared significantly improved only upon BIL. Moreover, as shown in Table 3, UNI_D induced a statistically significant improvement in the motor UPDRS sub-scores of both ipsilateral (Δ% = median percentage improvement = 34.2) and contralateral side(Δ% = 80.1), while UNI_ND significantly reduced contralateral (Δ% = 54.6) but not ipsilateral symptoms (Δ% = 7.9).

**Table 3:** Motor Unified Parkinson’s Disease Rating Scale scores (median and range) related to the side of the body ipsilateral and contralateral to the different unilateral subthalamic nucleus (STN) stimulation conditions for the group with a dominant STN (DOM) and the group without a dominant STN (NDOM).

		OFF	UNI_D	Δ%	OFF	UNI_ND	Δ%
DOM (*n* = 6)	Ipsilateral body side	15.5 (12.5–29.0)	9.3 (5.5–18.5)	34.2* (26.8–64.5)	21.8 (14.0–24.0)	17.0 (7.5–23.5)	7.9 (0.0–63.4)
	Contralateral body side	21.8 (14.0–24.0)	4.0 (3.0–9.0)	80.1* (35.7–85.4)	15.5 (12.5–29.0)	8.3 (3.5–11.5)	54.6* (39.3–77.4)

		**OFF**	**UNI_R**	**Δ%**	**OFF**	**UNI_L**	**Δ%**

NDOM (*n* = 4)	Ipsilateral body side	21.5 (18.5–22.0)	19.0 (13.5–23.0)	11.8 (−4.5 to 27.0)	24.0 (20.5–28.5)	25.3 (19.5–28.5)	1.0 (−13.0 to 4.9)
	Contralateral body side	24.0 (20.5–28.5)	13.5 (13.0–17.0)	42.3* (26.1–50.9)	21.5 (18.5–22.0)	8.8 (1.0–13.0)	60.2* (38.1–94.6)

*Δ%, percentage improvement with respect to basal (OFF) condition; OFF, basal condition (medication off-bilateral stimulation off); UNI_D, unilateral stimulation of dominant STN; UNI_ND, unilateral stimulation of non-dominant STN; UNI_R, unilateral stimulation of right STN; UNI_L, unilateral stimulation of left STN*.

Symbol “*” indicates statistically significant difference (*p* < 0.05) between OFF condition and a specific unilateral stimulation condition.

By contrast, in the NDOM group only bilateral stimulation significantly improved motor UPDRS score and sub-scores (see Table 2, A), while unilateral stimulation of right (UNI_R) and left (UNI_L) STN reduced PD motor symptoms in a similar manner, but not significantly. Moreover, as shown in Table 3, unilateral stimulation significantly improved contralateral motor symptoms (UNI_R: Δ% = 42.3; UNI_L: Δ% = 60.2) but not ipsilateral scores (UNI_R: Δ% = 11.8; UNI_L: Δ% = 1.0).

#### Effects of STN Stimulation on Gait

Table 2 (B–E) summarized the results emerged from the gait analysis in the four STN stimulation conditions.

**Figure 2:**
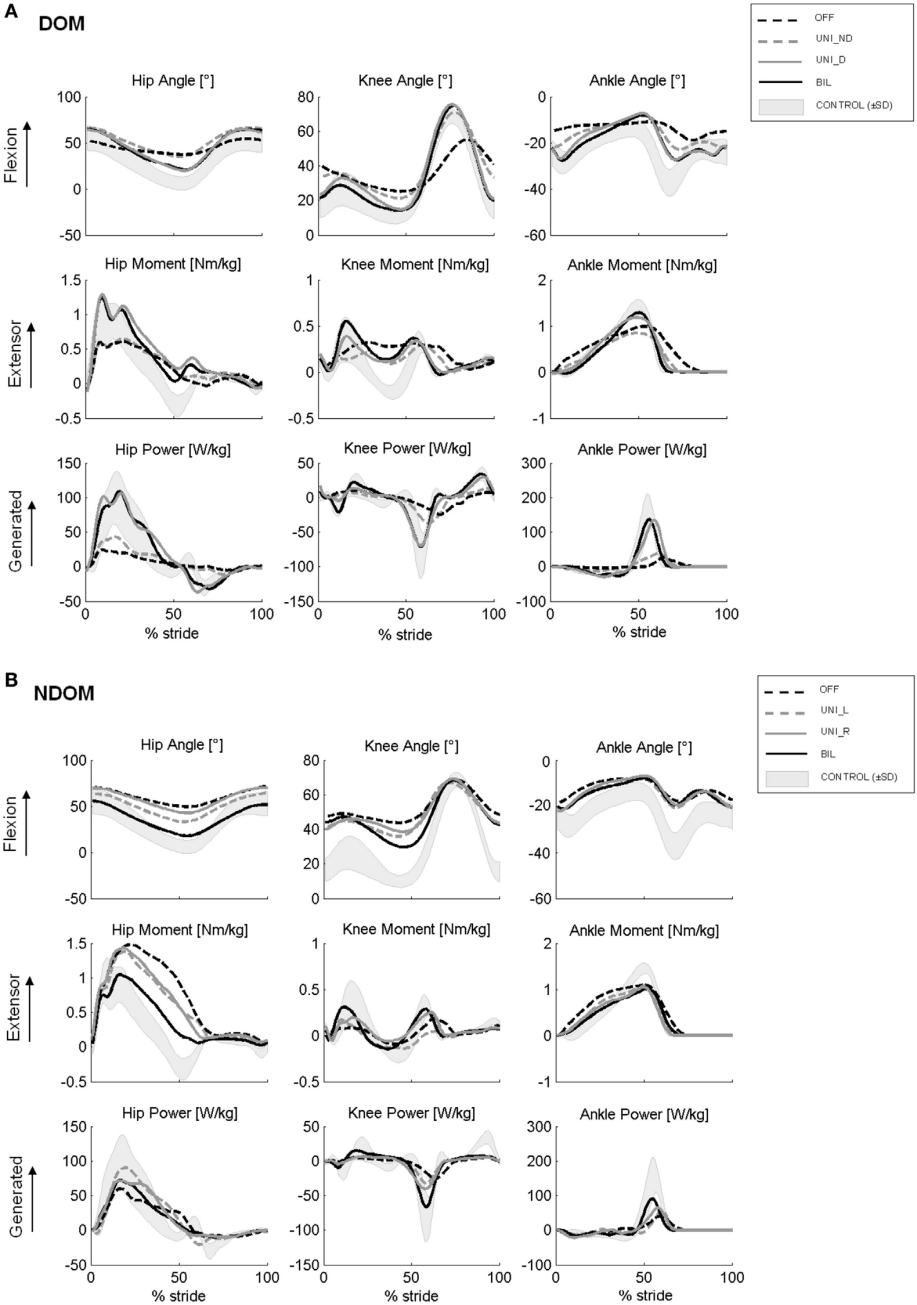
Kinematic and kinetic profiles (within-subject mean curves) from representative Parkinson’s disease subjects with dominant subthalamic nucleus (STN) **(A)** and without dominant STN **(B)** in the different STN stimulation conditions. From panels **(A)**, note that unilateral stimulation of the dominant STN (UNI_D) improves all profiles more than UNI_ND and similarly to bilateral stimulation (BIL). From panels **(B)**, note that unilateral stimulation of right (UNI_R) and left STN (UNI_L) produce similar improvement lower than that generated by bilateral simulation (BIL).

**Figure 3:**
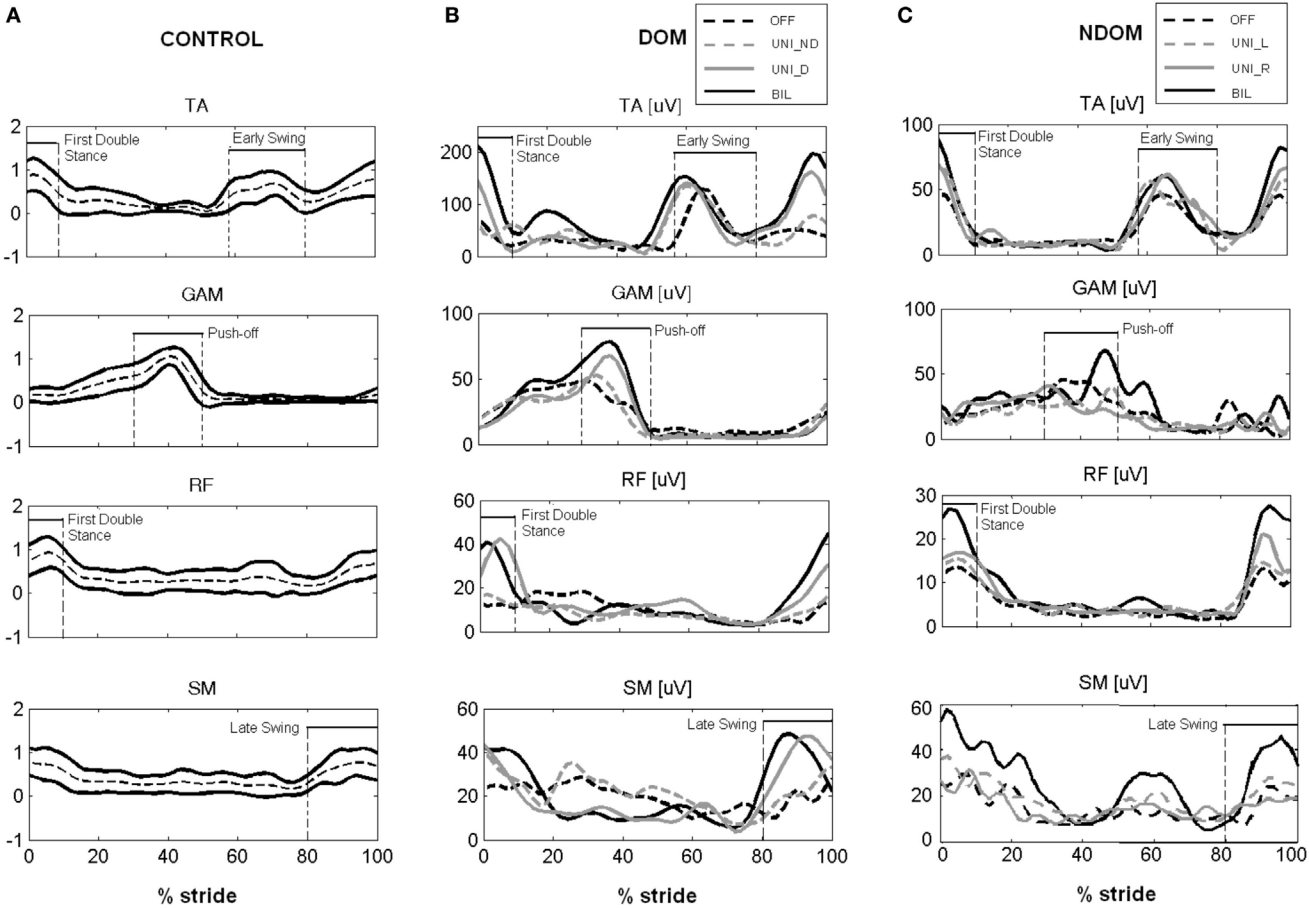
**(A)** Electromyographic (EMG) linear envelopes for control subjects. Intersubject mean curve (dashed line) ± SD band (bold lines). Amplitude is normalized to 95th percentile of control group. **(B,C)** Representative examples of EMG linear envelopes (within-subject mean) from Parkinson’s disease patients with dominant subthalamic nucleus (STN) **(B)** and without dominant STN **(C)** in the different conditions.

In OFF condition, both DOM and NDOM patients showed similar gait characteristics. In particular, both PD groups adopted statistically significant lower velocity, shorter stride length and longer stance time, with respect to healthy control subjects (Table 2, B). Both groups showed lower ROM of hip, knee and ankle joints (Table 2, C; Figure 2), while only DOM group demonstrated statistically significant alterations of gait kinetics, in particular lower moment peak at the ankle and lower power peak at the hip and ankle joints (Table 2, D; Figure 2). Visual analysis of EMG signals revealed consistent anomalies in the activation pattern of tested muscles in both DOM and NDOM groups with respect to healthy controls (see Figures 3A–C). In particular, PD patients showed: (i) a reduced or absent activity of RF during first double stance, (ii) anticipated and reduced or absent activation peak of GAM during push-off phase, and (iii) a reduced activity of TA during early swing and, at a larger extent, during first double stance.

In the DOM group, both BIL and UNI_D stimulation significantly increased, to a similar extent, gait speed, stride length (Table 2, B), the angular displacements (ROM) of the three main lower limb joints (Table 2, C; Figure 2A) and the peaks of moment and power at the ankle joint (Table 2, D; Figure 2A). Conversely, no statistically significant differences between OFF and UNI_ND conditions were noticed in any aforementioned variables (Table 2, B–D; Figure 2A). With reference to muscles activity, both BIL and UNI_D similarly improved the EMG activation pattern of distal and, to a lower extent, of proximal leg muscles (Table 2, E). In particular (see also Figure 3B), upon BIL and UNI_D, the activation burst of TA typically present around ground contact, at the beginning of the first double stance, was restored, GAM muscle increased its EMG activity during push-off, while RF and SM recovered their activation burst during first double support and late swing, respectively. Conversely, no significant improvements were noticed in UNI_ND condition (Table 2, E; Figure 3B).

In the NDOM group (Table 2, B), unilateral and bilateral stimulation slightly increased gait speed and stride length, but a statistically significant improvement was noticed only upon BIL. Kinematic and kinetic variables were almost unaltered by STN stimulation, even though a trend toward an increase of hip angular displacement and ankle power peak was present (Table 2, C,D). Moreover, a shift of the kinematic and kinetic profiles toward normal templates was noticed for the three considered joints (Figure 2B). This trend was more pronounced upon bilateral than unilateral stimulation. Bilateral stimulation significantly increased the EMG activity of TA during the first double stance and GAM at push-off. In addition, a trend toward improved activation was noticed in RF during first double support and in SM during late swing, although non-statistically significant (Table 2, E; Figure 3C). Unilateral right and left stimulation did not generate significant improvements (Table 2, E; Figure 3C).

In both DOM and NDOM group, none of the aforementioned variables showed the significant asymmetries between ipsilateral and contralateral sides that emerged from clinical assessment.

#### Location of the Active Contacts and Stimulation Parameters

As shown in Table 4, no differences were found between DOM and NDOM groups in either the location of the active contacts or the electric parameters of stimulation.

**Table 4:** Comparison of the location of the active contacts and of the stimulation parameters between patients with dominant (DOM) and without dominant (NDOM) subthalamic nucleus (STN), between the dominant (D_STN) and the not-dominant (ND_STN) STN in the DOM group and between the right (R_STN) and the left (L_STN) STN in the NDOM group.

	DOM group (*n* = 6)	NDOM group (*n* = 4)	*p*-Value	DOM D_STN	DOM ND_STN	*p*-Value	NDOM R_STN	NDOM L_STN	*p*-Value
**Active contacts position^a^**									
Lateral (mm)	11.8 ± 0.6	11.6 ± 0.7	1.000	12.0 ± 0.0	11.5 ± 0.8	0.157	11.6 ± 0.8	11.6 ± 0.8	1.000
Antero-posterior (mm)	−1.3 ± 0.9	−1.7 ± 0.9	0.172	−0.9 ± 1.0	−1.7 ± 0.7	0.078	−1.1 ± 1.0	−2.2 ± 0.4	0.141
Vertical (mm)	−1.3 ± 1.3	−2.1 ± 1.3	0.291	−1.0 ± 1.7	−1.6 ± 0.7	0.500	−1.6 ± 1.4	−2.6 ± 1.2	0.273
**Parameters of stimulation**									
Voltage (V)	3.1 ± 0.3	3.1 ± 0.5	0.944	3.1 ± 0.3	3.2 ± 0.3	0.655	3.1 ± 0.6	3.1 ± 0.5	0.655
Pulse width (μs)	67.5 ± 13.6	78.8 ± 15.6	0.257	70.0 ± 15.5	65.0 ± 12.2	0.317	82.5 ± 15.0	75.0 ± 17.3	0.317
Frequency (Hz)	144.2 ± 19.3	141.9 ± 15.3	0.273	145.8 ± 22.2	142.5 ± 17.8	0.524	143.8 ± 18.9	140.0 ± 13.5	0.461

*^a^Location of the active contacts in relation to the midcommissural point*.

*D_STN, dominant STN; ND_STN, non dominant STN; R_STN, right STN; L_STN, left STN*.

*Values are mean ± SD*.

Moreover, no statistically significant differences were found in the electrodes location and stimulation parameters between the dominant and non-dominant side of stimulation for DOM group, and between right and left stimulation sides for NDOM group (Table 4).

## Discussion

In our study, we used the method described by Castrioto et al. (25) to verify if, in a group of PD patients treated with bilateral STN-DBS, it was possible to identify a subgroup who presented the dominance of one STN, and to quantitatively assess the effect of the unilateral stimulation of the dominant STN using a kinematic, kinetic and EMG analysis of overground walking.

Indeed, the effects of unilateral STN-DBS on gait are not fully understood (33). While both improvements and worsening of gait performances were reported at individual level in medication-on condition (34, 35), in medication-off condition significant improvements induced by unilateral STN stimulation have been documented in gait initiation (8) and steady-state walking (16–18, 21, 36–39). Most of these studies analyzed gait improvement through UPDRS gait item (16, 17, 39) or spatiotemporal parameters (16, 18, 21, 36, 39). Only three studies analyzed the effect of unilateral stimulation on more specific aspects of gait, such as spatiotemporal parameters (24), lower limb joints kinematics (37) and EMG activity of leg muscles (38). In one of these studies it was also hypothesized a dominant role of the right side motor network for gait control (24). To the best of our knowledge, no study related to multifactorial analysis of gait which combines kinematics, kinetics and EMG data has been performed so far for a complete evaluation of the effects of unilateral stimulation of dominant and non-dominant STN.

In our study, we found that 60% of patients (6/10) showed a dominant STN, whose stimulation determined a 55.9% improvement in motor UPDRS score with respect to the off stimulation condition, slightly (but not statistically) inferior to what observed with bilateral STN stimulation. In one patient the dominant STN was ipsilateral to the most affected body side. Moreover, the motor improvement due to the dominant STN stimulation was significant both on the contralateral and on the ipsilateral body side. By contrast, the unilateral stimulation of the non-dominant STN did not provide any statistically significant improvements in motor UPDRS score and sub-scores, despite the similar location of the active contacts and the comparable electric stimulation parameters. These findings, in turn, confirm those obtained by Castrioto et al. (25) and seem to support the existence of the “dominant-STN phenomenon” in a subgroup of PD subjects.

By contrasts, it was not possible to detect a significant improvement on clinical gait score, as measured by UPDRS item 29, for dominant-STN stimulation, probably for the small sample size and for the relative poor sensibility of the UPDRS gait score. These limitations are partly overcome by the multifactorial gait analysis performed in this study, which instead revealed significant ameliorations of walking provided by the dominant-STN stimulation. In particular, the analysis of the spatiotemporal gait variables demonstrated that both bilateral and unilateral stimulation of the dominant STN significantly increased gait speed and stride length, while no significant changes were observed in cadence. This is an interesting result which suggests that, similarly to bilateral STN-DBS, also unilateral DBS of the dominant STN can improve walking velocity by increasing stride length, typically shortened in PD (40), but not cadence, whose control has been demonstrated to be intact in these subjects (41). The effect of unilateral STN stimulation on stride length was analyzed also by Lizarraga et al. (24), who found that right-sided stimulation provided an improvement similar to bilateral stimulation and significantly higher than left-sided stimulation. This finding is in accordance with the present results, which show that four out of six DOM patients of our sample have a dominant right STN.

The angular displacements of the three main lower limb joints and the peaks of moment and power at the ankle joint were also similarly increased by both bilateral and unilateral dominant-STN stimulation. Finally, the EMG activation pattern, mainly of distal muscles, was improved by both these stimulation modalities. By contrast, the unilateral stimulation of the non-dominant STN in the DOM group and the unilateral stimulation of right or left STN in the NDOM group, failed to reveal a significant effect on the above reported gait parameters compared with OFF condition.

Analyzing the effect of unilateral STN stimulation on all gait variables, we did not observe any significant difference between the ipsilateral and contralateral body side, contrarily to what was found for motor UPDRS score; this could indicate that the gait improvement observed with the unilateral stimulation of the dominant STN is probably due to a bilateral effect on the locomotor system.

The anatomical explanation for the bilateral effect of unilateral STN stimulation could be the presence of several cross-connections between the basal ganglia and the cortical areas (42, 43) and between the basal ganglia and the peduncolopontine nucleus (PPN), component of the mesencephalic locomotor reticular region (44, 45).

Subthalamic nucleus receives inhibitory inputs from the ipsi- and contralateral external globus pallidus (GPe) (46) and excitatory inputs from the ipsilateral premotor and motor cortex (47) and from the ipsi- and contralateral parafascicular nucleus of the thalamus (Pf-Th) (46, 48). Furthermore, it projects itself excitatory inputs to the Pf-Th (46).

The interconnection of the two STN is demonstrated by the recording of an increase in contralateral STN neuronal activity for the unilateral STN stimulation (49, 50). Moreover, unilateral STN stimulation seems also capable to determine changes in bursting an oscillatory activity of contralateral STN neurons, in relation for example to the presence or absence of stimulation-induced dyskinesias (51).

The existence of bilateral connections of basal ganglia nuclei is also demonstrated by the observation that unilateral STN-DBS produces bilateral cerebral blood flow responses (rCBF) in cortical and cerebellar regions (52–54), and that these rCBF changes do not differ for ipsi- or contralateral STN stimulation (54).

Moreover, recently some authors demonstrated the presence of an interhemispheric functional connectivity studying the STN-Local Field Potential coherence (55–58).

The effects of STN-DBS on gait are likely to be related to the interconnection between the STN and the PPN (59). Recently, Neagu et al. (60) demonstrated the presence of ipsilateral PPN long latency polyphasic potentials (40–45 ms) in patients unilaterally stimulated in the STN. They argued that these potentials could be the result of the activation of fibers or other structures near the PPN, probably through a polysynaptic pathway on the basis of their long latency. This pathway could be represented by ipsilateral projections from internal globus pallidus (GPi) to PPN (61) or most likely by connections between the precentral and premotor cortex, the red nucleus and the PPN (62, 63).

Furthermore, the recent advent of directional leads capable to focus the stimulation field in a specific horizontal direction (64), combined with instrumented gait analysis method, might provide insights on the role of specific basal ganglia regions in the motor control and potentially facilitate the individual tailoring of STN-DBS modality.

One of the main limitations of our study is represented by the small sample size, partially offset by the use of quantitative methods of analysis. Moreover, the low number of patients does not allow the identification of predictive factors for the presence of a dominant STN.

Further studies are needed to identify through functional or imaging techniques the presence of a dominant STN before surgery.

In conclusion, although caution must be taken given the low number of tested subjects, our results seemed to demonstrate the presence of a dominant STN in more than 50% of our sample of PD patients bilaterally implanted for STN-DBS. In these patients, the unilateral stimulation of the dominant STN was capable to determine a clinical improvement, measured with UPDRS score, similar to what observed with bilateral STN stimulation. Importantly, the use of more sophisticated methods such as multifactorial quantitative gait analysis, suggested a role of the dominant STN also on axial symptoms involved in walking function, whose disturbances play a relevant role in determining disability and changes in quality of life (26, 28) of PD patients. If it was possible to identify the PD patients with a dominant STN before surgery, it could be indicated for them an unilateral implant, to minimize the surgical risks, to reduce the side effects due to bilateral DBS and the duration and costs of the procedure.

## Ethics Statement

This study was carried out in accordance with the recommendations of Ethics Committee of the CTO Hospital (Turin, Italy) with written informed consent from all subjects. All subjects gave written informed consent in accordance with the Declaration of Helsinki. The protocol was approved by the Ethics Committee of the CTO Hospital (Turin, Italy).

## Author Contributions

MGR, LL, MML, and MF designed the study. MGR, LL, and MML recruited the subjects and MML performed the surgery. MGR and MF participated in data acquisition. IC processed and analyzed the data. MGR, MF, LL, MML, and IC contributed to data interpretation. MGR and IC drafted the manuscript. MF, LL, and MML participated in the critical revision process. All the authors approved the manuscript.

## Conflict of Interest Statement

The authors declare that the research was conducted in the absence of any commercial or financial relationships that could be construed as a potential conflict of interest.
